# Association between delayed bedtime and sleep-related problems among community-dwelling 2-year-old children in Japan

**DOI:** 10.1186/s40101-015-0050-x

**Published:** 2015-03-13

**Authors:** Shingo Kitamura, Minori Enomoto, Yuichi Kamei, Naoko Inada, Aiko Moriwaki, Yoko Kamio, Kazuo Mishima

**Affiliations:** Department of Psychophysiology, National Institute of Mental Health, National Center of Neurology and Psychiatry, 4-1-1 Ogawa-Higashi, Kodaira, Tokyo 187-8553 Japan; Graduate School of Education, The University of Tokyo, 7-3-1 Hongo, Bunkyo-ku, Tokyo 113-8654 Japan; Support Room for Students with Special Needs, Tokyo Gakugei University, 4-1-1, Nukui Kitamachi, Koganei, Tokyo 184-8501 Japan; Department of Child and Adolescent Mental Health, National Institute of Mental Health, National Center of Neurology and Psychiatry, 4-1-1 Ogawa-Higashi, Kodaira, Tokyo 187- 8553 Japan

**Keywords:** Sleep habits, Sleep problems, Toddlers, Prevalence, Cross-sectional study

## Abstract

**Background:**

Although delayed sleep timing causes many socio-psycho-biological problems such as sleep loss, excessive daytime sleepiness, obesity, and impaired daytime neurocognitive performance in adults, there are insufficient data showing the clinical significance of a ‘night owl lifestyle’ in early life. This study examined the association between habitual delayed bedtime and sleep-related problems among community-dwelling 2-year-old children in Japan.

**Methods:**

Parents/caregivers of 708 community-dwelling 2-year-old children in Nishitokyo City, Tokyo, participated in the study. The participants answered a questionnaire to evaluate their child’s sleep habits and sleep-related problems for the past 1 month.

**Results:**

Of the 425 children for whom complete data were collected, 90 (21.2%) went to bed at 22:00 or later. Children with delayed bedtime showed significantly more irregular bedtime, delayed wake time, shorter total sleep time, and difficulty in initiating and terminating sleep. Although this relationship indicated the presence of sleep debt in children with delayed bedtime, sleep onset latency did not differ between children with earlier bedtime and those with delayed bedtime. Rather, delayed bedtime was significantly associated with bedtime resistance and problems in the morning even when adjusting for nighttime and daytime sleep time.

**Conclusions:**

Even in 2-year-old children, delayed bedtime was associated with various sleep-related problems. The causal factors may include diminished homeostatic sleep drive due to prolonged daytime nap as well as diurnal preference (morning or night type) regulated by the biological clock.

## Background

Approximately 25% of children are reported to experience some type of sleep problem [1]. The most commonly reported sleep-related problems are sleep loss and subsequent daytime dysfunction. Sleep-deprived children show the long-lasting effects of sleep loss on a wide range of mental and physical functions, including declined neurocognitive performance, memory loss, anxiety, depression, and obesity [1-6]. These effects seem to be more critical in younger age. For example, longitudinal study showed that children with short sleep (<10 h) before age at 3.5 years were 2.5-fold more likely to show the externalized problems in age at school entry compared with children with 11 h of sleep, even if they slept longer later [7].

In daily life, most cases of sleep loss are caused by delayed bedtime (BT). Delayed BT in infants and toddlers is common in developed countries [8,9] including Japan, where the number of such cases has been increasing for some time. A survey of approximately 10,000 preschool children has shown that the rate of children who go to bed at 22:00 or later increased linearly over the period from 1980 to 2000 [10]. Children with delayed BT often exhibit various sleep-related problems, including BT resistance and morning irritability [8,11-14]. Because children’s sleep-related problems can intensify parent’s depressive state and consequently reduce the quality of family functioning [15-18], they are a major public health concern that needs to be urgently addressed.

Even when children regularly have delayed BT, they are generally not allowed to sleep late in the morning so as to fit in with their parents’ schedule. Consequently, compared with children with earlier BT, children with delayed BT have shorter total sleep time and longer daytime nap time to compensate for the sleep debt even in toddlers [19]. However, excessive daytime napping diminishes homeostatic sleep drive, and reduced sleep propensity during conventional sleep hours may lead to recurring delays in BT and persistent sleep debt. A negative cycle between habitually delayed BT, sleep loss, and daytime napping is thought to be a frequent cause of BT resistance, difficulty with morning awakening, and related negative mood and poor appetite [20]. However, the relationships between sleep habits and sleep-related problems at BT and in the morning in children with delayed BT have yet to be elucidated.

To this end, this study aimed to investigate the relationship between delayed BT, sleep habits, and sleep-related problems at night and in the morning in community-dwelling 2-year-old children.

## Methods

### Participants

Potential participants were parents/caregivers of 1,570 community-dwelling 2-year-old children born between November 2006 to October 2007 and living in Nishitokyo City, Tokyo (population approximately 197,000). Parents/caregivers of 829 out of 1,570 children who visited 24-month-old health checkup voluntarily were asked to complete a survey on their child’s sleep habits and sleep-related problems. The survey was conducted between November 2008 and October 2009. Children whose age in months, sex, and birth order which were not provided in the survey were excluded from the study.

Written consent was obtained from all participants, who were provided with explanations about the study and its purposes. The study was approved by the Institutional Review Board of the National Center of Neurology and Psychiatry, Japan.

### Measures

To assess children’s sleep habits and sleep-related problems, parents/caregivers were asked to answer 32 originally developed questionnaire items and demographical information after receiving an explanation of the study at the checkup. Those who did not have time to fill in the questionnaire at the checkup venue completed the questionnaire at home and returned it by mail.

Sleep habits were evaluated using nine questionnaire items including bed sharing, child’s BT, BT irregularity (on average, 90 min per week), sleep onset latency (SOL; minutes to falling asleep at night), number and duration of nocturnal awakenings (WASO), wake time (WT), number of daytime sleeps, and total daytime sleep time (NAP) for the last 1 month. From these items, the following variables were extracted: sleep onset time (SOT; BT + SOL), time in bed (TIB; elapsed interval from BT to WT), total night sleep time (TST; TIB − (SOL + WASO)), and sleep efficiency (SE; ratio of TST to TIB). Answers were excluded if the values were considered highly unlikely or extreme (for example, sleep and wake times appeared to be reversed, total sleep time was less than 4 h, or total sleep time was more than 22 h).

Sleep-related problems were evaluated using the remaining 23 items. Part of these items was depicted from the Children’s Sleep Habits Questionnaire [21]. Although these items included BT behavior (4 items), behavior occurring during sleep (12 items), problems on morning awakening (5 items), and daytime sleepiness (2 items) in the last 1-month period, 6 items that are thought to be associated with delayed BT were used in the analysis: ‘struggles at bedtime’, ‘wakes up in a negative mood’, ‘has a hard time getting out of bed’, ‘takes a long time to be alert’, ‘has poor appetite in the morning’, and ‘seems very sleepy.’ Each item was evaluated using a 3-point Likert scale (1: rarely [never or once per week], 2: sometimes [two to four times per week], and 3: usually [five or more times per week]). We analyzed these items individually because they were not made as composite items.

### Statistical analysis

One-way analysis of variance (ANOVA) was used for comparisons of sleep habits, except BT irregularity, across the BT groups. Multiple comparisons using Bonferroni’s method were then conducted for those variables showing main effects. Chi-square tests were used to compare BT irregularity and the incidence of sleep problems across the BT groups with Bonferroni corrections.

Hierarchical logistic regression was carried out with the incidence of each sleep-related problem (present two or more times per week) as the dependent variable and BT as the independent variables in model 1 (crude model). In model 2, demographic variables (age in months, sex, birth order) were entered as covariates. In model 3, demographic variables and TST, NAP, and BT irregularity were entered as covariates. BT was divided into four categories based on the clock time, with the category having the earliest BT (before 21:00) regarded as the reference category. Wald’s test was used to calculate odd ratios (ORs).

IBM SPSS Statistics version 21.0 (IBM Corporation) was used for statistical analysis, setting 5% for all critical probability levels. Data are expressed as means ± standard deviation (SD).

## Results

Responses were received from parents/caregivers of 708 children (survey response rate, 85.4%). As the first 246 of parents/caregivers were asked to answer only sleep habits and 37 out of the remaining 462 children for whom data were incomplete, data were analyzed for 425 children: 204 boys (48.0%), 221 girls (52.0%), mean age 24.1 months (SD, 1.0 month; range, 22 to 30 months), 259 first-borns (60.9%), 134 second-borns (31.5%), and 32 third or later borns (7.5%).

To reveal associations between delayed BT, sleep habits, and sleep-related problems, children were divided into four groups according to their BT: BT before 21:00 (up to 2100hG; *n* = 121, 28.5%), BT between 21:00 and 21:29 (2100hG; *n* = 137, 32.2%), BT between 21:30 and 21:59 (2130hG; *n* = 77, 18.1%), and BT after 22:00 (2200h or later G; *n* = 90, 21.2%). No significant differences were seen in mean age (*F*(3,421) = 0.277, *p* = 0.842) between the four groups; however, the male-to-female ratio (*χ*^2^(3) = 13.696, *p* = 0.003) and the proportion of birth order (*χ*^2^(3) = 15.272, *p* = 0.018) were significantly different among four groups. Boys showed later bedtime than girls, and first-born children showed later bedtime than children with later birth order.

### Sleep habits

Of 425 parent-child units, 423 (99.5%) shared a bedroom. Mean BT was 21.2 (0.9) and ranged from 18:00 to 01:00. BT was after 22:00 in 90 children (21.2%) and was irregular in 178 children (41.9%). Mean SOL, TST, WT, and NAP were 29.9 ± 15.7 min, 9.4 ± 0.9 h, 7.2 ± 0.9 o’clock, and 1.8 ± 0.7 h, respectively.

Table 1 shows sleep habits in the four different BT groups. WT was delayed significantly, by 1.3 h on average, with delayed BT from the up to 2100hG to the 2200h or later G (*F*(3,421) = 50.859, *p* < 0.001), with a significant difference seen between all groups on multiple comparison analysis (all corrected *p* < 0.01). With a delayed BT, TIB (*F*(3,421) = 26.125, *p* < 0.001) and TST (*F*(3,421) = 22.193, *p* < 0.001) were significantly decreased, on average by 01.0 h, in the 2200h or later G group compared with the up to 2100hG. The up to 2100hG had the longest TIB and TST among the groups (all corrected *p* < 0.01), whereas the 2200h or later G had the shorter TIB and TST compared to the up to 2100hG and 2100hG (all corrected *p* < 0.01). No significant differences in TIB and TST were found between 2130hG and 2200h or later G.

**Table 1:** **Sleep habits by bedtime**

	**Whole**	**Up to 2100hG**	**2100hG**	**2130hG**	**2200h or later G**	***F / χ*** ^***2***^	***p***
Number of participants	425	121	137	77	90		
Bedtime (BT) (h)	21.2 ± 0.9	20.2 ± 0.5	21.0 ± 0.1	21.5 ± 0.1	22.4 ± 0.6	649.293	<0.001
Sleep onset latency (SOL) (min)	29.9 ± 15.7	29.3 ± 14.4	30.7 ± 16.6	30.1 ± 14.7	29.1 ± 16.7	0.266	0.850
Sleep onset time (SOT) (h)	21.7 ± 0.9	20.7 ± 0.5	21.5 ± 0.3	22.0 ± 0.2	22.9 ± 0.6	461.334	<0.001
Wake time (WT) (h)	7.2 ± 0.9	6.7 ± 0.6	7.0 ± 0.8	7.4 ± 0.6	7.9 ± 1.0	50.859	<0.001
Time in bed (TIB) (h)	10.0 ± 0.8	10.5 ± 0.7	10.0 ± 0.8	9.8 ± 0.6	9.5 ± 1.0	26.125	<0.001
Total night sleep time (TST) (h)	9.4 ± 0.9	9.9 ± 0.7	9.4 ± 1.0	9.2 ± 0.8	8.9 ± 1.0	22.193	<0.001
Wake after sleep onset (WASO) (min)	6.6 ± 21.4	5.3 ± 17.3	7.0 ± 27.1	7.7 ± 18.1	7.0 ± 19.6	0.247	0.864
Sleep efficiency (SE) (%)	93.9 ± 4.6	94.5 ± 3.3	93.7 ± 5.5	93.6 ± 4.4	93.7 ± 4.6	1.040	0.375
Total daytime sleep time (NAP) (min)	1.8 ± 0.7	1.6 ± 0.5	1.8 ± 0.8	1.9 ± 0.6	2.1 ± 0.6	7.078	<0.001
Bedtime irregularity (%)	41.9%	33.1%	38.7%	49.4%	52.2%	10.163	0.017

NAP was significantly increased by an average of 0.4 h with a delayed BT from the up to 2100hG to the 2200h or later G (*F*(3,421) = 7.078, *p* < 0.001). In addition, the later BT was, the higher the proportion of children with irregular BT (*χ*^2^(3) = 10.163, *p* = 0.017), and this was the case for 52.2% of the children in the 2200h or later G.

There were no significant differences in SOL (*F*(3,421) = 0.266, *p* = 0.850), WASO (*F*(3,421) = 0.247, *p* = 0.864), or SE (*F*(3,421) = 1.040, *p* = 0.375) between the four BT groups.

### Sleep-related problems

Table 2 shows sleep-related problems for the four BT groups. Of the six questionnaire items on sleep-related problems, significant differences in prevalence were observed for ‘struggles at bedtime’ (*χ*^2^(3) = 23.017, corrected *p* < 0.001), ‘has a hard time getting out of bed’ (*χ*^2^(3) = 21.680, *p* < 0.001), ‘takes a long time to be alert’ (*χ*^2^(3) = 12.424, *p* < 0.001), and ‘has poor appetite in the morning’ (*χ*^2^(3) = 20.516, *p* = 0.001), but not for ‘wakes up in a negative mood’ (*χ*^2^(3) = 8.513, corrected *p* = 0.219) and ‘seems very sleepy’ (*χ*^2^(3) = 1.841, corrected *p* = 1.000).

**Table 2:** **Incidence of sleep-related problems by bedtime**

	**Whole**		**Up to 2100hG**		**2100hG**		**2130hG**		**2200h or later G**		***χ*** ^***2***^	***p***
	***n***	**(%)**	***n***	**(%)**	***n***	**(%)**	***n***	**(%)**	***n***	**(%)**		
Struggles at bedtime	145	(34.1)	25	(20.7)	48	(35.0)	25	(32.5)	47	(52.2)	23.017	<0.001
Wakes up in negative mood	111	(26.1)	24	(19.8)	31	(22.6)	24	(31.2)	32	(35.6)	8.513	0.037
Hard time getting out of bed	47	(11.1)	4	(3.3)	11	(8.0)	12	(15.6)	20	(22.2)	21.680	<0.001
Takes long time to be alert	52	(12.2)	6	(5.0)	15	(10.9)	14	(18.2)	17	(18.9)	12.424	<0.001
Has poor appetite in the morning	76	(17.9)	11	(9.1)	18	(13.1)	21	(27.3)	26	(28.9)	20.516	<0.001
Seems very sleepy	89	(20.9)	26	(21.5)	32	(23.4)	12	(15.6)	19	(21.1)	1.841	0.606

Logistic regression analysis of the four items revealed that there were significant associations between BT and sleep-related problems (Table 3). These relationships were observed even after adjusting for TST, NAP, BT irregularity, as well as demographic variables (age in months, sex, and birth order). In fully adjusted model (model 3), the ORs between the 2200h or later G and up to 2100hG groups yielded adjusted ORs of 2.9 (95% CI: 1.5 to 5.6, *p* = 0.002) for ‘struggles at bedtime’, 5.9 (95% CI: 1.7 to 20.0, *p* = 0.004) for ‘has a hard time getting out of bed’, 4.1 (95% CI: 1.4 to 12.0, *p* = 0.010) for ‘takes a long time to be alert’, and 3.8 (95% CI: 1.6 to 8.8, *p* = 0.002) for ‘has poor appetite in the morning’. Thus, children going to bed 22:00 h or later was 3- to 6-fold more likely to experience sleep-related problems than the children going to bed 21:00 h or earlier. In addition, compared with the up to 2100hG, the 2100hG had significant ORs of 1.8 (95% CI: 1.0 to 3.3, *p* = 0.049) for ‘struggles at bedtime’ and the 2130hG had significant ORs of 4.3 (95% CI: 1.3 to 14.8, *p* = 0.020) for ‘has a hard time getting out of bed,’ 3.9 (95% CI: 1.4 to 11.3, *p* = 0.011) for ‘takes a long time to be alert,’ and 3.6 (95% CI: 1.5 to 8.4, *p* = 0.003) for ‘has poor appetite in the morning’.

**Table 3:** **Multivariate logistic regression analysis predicting the incidence of sleep-related problems**

	**Struggles at bedtime**		**Wakes up in negative mood**		**Hard time getting out of bed**		**Takes a long time to be alert**		**Has bad appetite in the morning**	
	**OR**	**(95% CI)**	**OR**	**(95% CI)**	**OR**	**(95% CI)**	**OR**	**(95% CI)**	**OR**	**(95% CI)**
Model 1 (unadjusted)										
2100hG	**2.1**	(1.2-3.6)*	1.2	(0.6-2.2)	2.6	(0.8-8.2)	2.4	(0.9-6.3)	1.5	(0.7-3.3)
2130hG	1.8	(1.0-3.5)	1.8	(0.9-3.5)	**5.4**	(1.7-17.4)**	**4.3**	(1.6-11.6)**	**3.7**	(1.7-8.3)**
2200h or later G	**4.2**	(2.3-7.7)**	**2.2**	(1.2-4.2)*	**8.4**	(2.7-25.5)**	**4.5**	(1.7-11.8)**	**4.1**	(1.9-8.8)**
Model 2 (adjusted for age, gender)										
2100hG	**2.0**	(1.1-3.5)*	1.1	(0.6-2.0)	2.6	(0.8-8.5)	2.3	(0.9-6.2)	1.5	(0.7-3.3)
2130hG	1.7	(0.9-3.2)	1.7	(0.9-3.3)	**5.5**	(1.7-18.0)**	**4.0**	(1.5-11.1)**	**3.6**	(1.6-8.1)**
2200h or later G	**3.6**	(1.9-6.7)**	**2.0**	(1.0-3.7)*	**8.2**	(2.6-25.4)**	**4.3**	(1.6-11.6)**	**3.8**	(1.7-8.4)**
Model 3 (adjusted for age, gender, total sleep time, nap time, bedtime irregularity)										
2100hG	**1.8**	(1.0-3.3)*	0.9	(0.5-1.7)	2.1	(0.6-7.1)	2.2	(0.8-6.0)	1.5	(0.7-3.4)
2130hG	1.4	(0.7-2.8)	1.4	(0.7-2.8)	**4.3**	(1.3-14.8)*	**3.9**	(1.4-11.3)*	**3.6**	(1.5-8.4)**
2200h or later G	**2.9**	(1.5-5.6)**	1.5	(0.7-3.0)	**5.9**	(1.7-20.0)**	**4.1**	(1.4-12.0)*	**3.8**	(1.6-8.8)**

OR: odd ratios, 95% CI: 95% confidence interval, *p < 0.05, **p < 0.01. Up to 2100h G was used as the reference category in each model. For a clearer explanation, all of statistically significant results were presented in bold.

## Discussion

This study investigated sleep habits and sleep-related problems in community-dwelling 2-year-old children with delayed BT. As BT was delayed, WT was significantly delayed; however, the magnitude of delay in WT was remarkably smaller than that in BT. As a result, TST was reduced by 1.0 h, from 9.9 h in the up to 2100hG to 8.9 h in the 2200h or later G. In contrast, with delayed BT, NAP was increased to compensate for this reduced TST. These results are comparable with previous studies, suggesting that children continuously accumulate sleep debt [8,11,12]. In addition, later bedtime was found in boys than girls and first-born children than children with later birth order. One possible factor of sex differences is that boys are vulnerable to the incidence of the developmental disorders, which are greatly likely to show the sleep problems (up to 80%) [22] including delayed bedtime. Regarding the birth order, Owens et al. [23] showed that children who lived with other children were nearly twice as likely to have early bedtimes compared to those without other children. Thus, increasing the number of family members may stabilize the sleep habits in children.

The study also revealed that sleep loss was not simply associated with earlier BT or earlier sleep onset in 2-year-old children with delayed BT. Compared with children with the earliest BT (up to 2100hG), NAP in children with the most delayed BT (2200h or later G) was only 0.5 h longer even though TST was reduced by 1.0 h. Despite a shorter TST, these children with delayed BT exhibited normal sleep latency and no clear signs of excessive daytime sleepiness (Tables 1 and 2). In adults, daytime arousal can be sustained by afternoon naps lasting only one third of the amount of sleep lost during the night before [24]. Similarly, the present results suggest that also in these children, extended NAP by approximately half of the sleep loss could at least partly compensate for sleep loss and consequently reduced homeostatic sleep pressure for the next night. In accordance with this, Fukuda and Sakashita [20] reported that, compared with kindergarten children with few daytime napping habits, nursery school children with a regular habit of daytime napping often went to bed later, with a high prevalence of sleep onset difficulties. Taken together, the present and previous findings indicate that delayed BT and sleep-related problems are caught in a negative loop involving reduced sleep pressure due to daytime napping causing BT struggles and delayed BT, and short sleep due to delayed BT resulting in problems in the morning and daytime napping.

On the other hand, children’s own biological traits could also contribute to their delayed BTs. Diurnal preferences, chronotypes, regulated by an individual’s biological clock largely determines natural sleep and behavioral timings in adults [25-28]. It is reasonable to assume that BTs are self-selected as early as age 2 because a distinct 24-h sleep-wake cycle is formed and sleep is consolidated at night in approximately 90% of children before the age of 1 year [29] and because approximately 50% of diurnal preferences are genetically determined [30-32]. The present study also indicates a possible linkage between diurnal preference and sleep-related problems in children. The logistic regression analysis revealed that delayed BT was significantly correlated with BT resistance and problems in the morning, even after adjusting for TST and NAP (model 3), suggesting that the correlation was not induced solely by insufficient sleep or reduction in sleep pressure due to daytime napping, but could be related to the preference for eveningness. Although the physiological mechanisms between eveningness and sleep problems are less understood, one possible factor is a breakfast composition. Children with poor tryptophan consumption at morning are likely to have difficulties in falling asleep and awakening, and to get angry and depressed [33,34]. Considering that tryptophan is a precursor for serotonin and melatonin, insufficient amount of tryptophan could lead to the malfunctions of circadian and mood regulations.

This study has several limitations. First, children’s sleep habits and sleep-related problems were evaluated using questionnaires answered by their parents/caregivers, which has less agreement with objective sleep measurement (that is, actigraphy). Second, as sleep questionnaire used in this study has not been validated, obtained scores do not imply the presence of any kinds of sleep disorders but simply reports the prevalence of sleep problems. Third, we could not provide the parent information such as socioeconomical states and behaviors around children’s sleep. Although it is well documented that parental behaviors, especially in mothers [35], could affect to their children’s sleep conditions, researches showed sleep interventions for young children have difficulties to improve children’s sleep problems [36], suggesting that sleep habits and sleep problems in children are mostly caused by their own characteristics.

Nevertheless, the findings of the present study revealed a large inter-individual variability in BT, TST, and NAP among 2-year-old children. Short sleep was a noticeable feature in children with delayed BT at a mere 2 years of age, and they also had a high prevalence of BT resistance, difficulty waking up, and other sleep-related problems. To treat children with sleep-related problems, it could be helpful to provide individualized therapy considering both sleep hygiene (daytime napping and BT) and chronobiological predispositions.

## Conclusions

The findings of this study demonstrated that delayed bedtime was associated with various sleep-related problems independent of sleep parameters such as total sleep time and daytime nap time. The causal factors may include diminished homeostatic sleep drive due to prolonged daytime nap as well as diurnal preference (morning or night type) regulated by the biological clock. Taking consideration of individual differences might be useful to improve children’s sleep-related problems.

## Abbreviations

BT: Bedtime
SOL: Sleep onset latency
WASO: Nocturnal awakenings
WT: Wake time
NAP: Total daytime sleep time
TIB: Time in bed
TST: Total night sleep time
SE: Sleep efficiency
OR: Odd ratio
CI: Confidence interval
